# Effects of absorbents on growth performance, blood profiles and liver gene expression in broilers fed diets naturally contaminated with aflatoxin

**DOI:** 10.5713/ajas.18.0870

**Published:** 2019-05-27

**Authors:** J. B. Liu, H. L. Yan, S. C. Cao, Y. D. Hu, H. F. Zhang

**Affiliations:** 1 School of Life Science and Engineering, Southwest University of Science and Technology, Mianyang, Sichuan 621010, China; 2 State Key Laboratory of Animal Nutrition, Institute of Animal Sciences, Chinese Academy of Agricultural Sciences, Beijing 100193, China; 3 Farm Animal Genetic Resources Exploration and Innovation Key Laboratory of Sichuan Province, Sichuan Agricultural University, Ya’an, Sichuan 625014, China

**Keywords:** Aflatoxin, Adsorbent, Broilers, Gene Expression, Growth Performance, Liver

## Abstract

**Objective:**

The study was conducted to evaluate the effects of the absorbent (a mixture of activated carbon and hydrated sodium calcium aluminosilicate) on growth performance, blood profiles and hepatic genes expression in broilers fed diets naturally contaminated with aflatoxin.

**Methods:**

A total of 1,200 one-day-old male chicks were randomly assigned to 6 treatments with 10 replicate cages per treatment. The dietary treatments were as follows: i) control (basal diets); ii) 50% contaminated corn; iii) 100% contaminated corn; iv) control+1% adsorbent; v) 50% contaminated corn+1% absorbent; vi) 100% contaminated corn+1% absorbent.

**Results:**

During d 1 to 21, feeding contaminated diets reduced (p<0.05) body weight (BW), average daily gain (ADG), and average daily feed intake (ADFI), but increased (p<0.05) feed-to-gain ratio (F/G). The absorbent supplementation increased (p<0.05) BW, ADG, and ADFI. There were interactions (p<0.05) in BW, ADG, and ADFI between contaminated corn and absorbent. Overall, birds fed 100% contaminated diets had lower (p<0.05) final BW and ADG, but higher (p<0.05) F/G compared to those fed control diets. The absorbent addition increased (p<0.05) serum albumin concentration on d 14 and 28 and total protein (TP) level on d 28, decreased (p<0.05) alanine transaminase activity on d 14 and activities of aspartate aminotransferase and alkaline phosphatase on d 28. Feeding contaminated diets reduced (p<0.05) hepatic TP content on d 28 and 42. The contaminated diets upregulated (p<0.05) expression of interleukin-6, catalase (CAT), and superoxide dismutase (SOD), but downregulated (p<0.05) glutathione S-transferase (GST) expression in liver. The absorbent supplementation increased (p<0.05) interleukin-1β, CAT, SOD, cytochrome P450 1A1 and GST expression in liver. There were interactions (p<0.05) in the expression of hepatic CAT, SOD, and GST between contaminated corn and absorbent.

**Conclusion:**

The results suggest that the naturally aflatoxin-contaminated corn depressed growth performance, while the adsorbent could partially attenuate the adverse effects of aflatoxin on growth performance, blood profiles and hepatic genes expression in broilers.

## INTRODUCTION

Aflatoxins (AFB), produced by *Aspergillus flavus* and *Aspergillus parasiticus*, are the most commonly occurring mycotoxins in poultry feed [1]. Aflatoxin B_1_ (AFB_1_), aflatoxin B_2_ (AFB_2_), aflatoxin G_1_ (AFG_1_), and aflatoxin G_2_ (AFG_2_) are the major forms of AFB, of which AFB_1_ is considered the most toxic [2]. The occurrence of AFB poses a great threat to broiler health because of its adverse effects on growth performance, immunity, intestinal morphology, blood profiles and hepatic functions [3–6]. A meta-analysis study indicated that AFB-contaminated diets caused 11% reduction in feed intake and daily weight gain in broilers, and the feed conversion ratio was also decreased by 6% [7].

It is therefore important to minimize the AFB exposure to prevent its detrimental effects. Recently, strategies, such as adsorption method, biodegradation and chemical treatment, have been proposed to alleviate the toxic effects of mycotoxins on livestock [3,8–9]. Among them, supplementation of adsorbent is the most suitable approach to detoxify mycotoxins-contaminated feed, because it can bind and immobilize mycotoxins to reduce toxic residues in feed and effectively prevent health risks in animals [10]. Hydrated sodium calcium aluminosilicate (HSCAS), as one of the adsorbents, has been proved to be effective in adsorbing AFB [11]. Activated carbon, another adsorbent, could improve body weight (BW) gain and feed conversion ratio of chickens fed AFB-contaminated diets [12]. However, the efficacy of a combination of the above two adsorbents has not been examined in broilers fed diets with AFB.

It is well documented that naturally contaminated diets were more toxic than diets containing purified mycotoxins. Nevertheless, few researches were conducted on the effect of naturally AFB-contaminated diets containing low levels of AFB_1_ and AFB_2_ on broilers [13,14]. The liver is a principal target organ of AFB and is also responsible for detoxification of AFB [15]. Previous study in broilers demonstrated that the liver would present pathological lesions and hepatic functions damage when the content of AFB_1_ was high (1 to 5 mg/kg) [4]. Notwithstanding, few literatures have examined the effect of corn naturally contaminated with low levels of AFB_1_ and AFB_2_ on the health and liver gene expression of broilers [5,16]. Furthermore, the effect of combined adsorbent on the response to naturally AFB-contaminated diets for broilers, with expected alleviation of toxicosis, has not yet been investigated. Therefore, the objective of this study was to determine the effect of the absorbent (a combination of activated carbon and HSCAS) on growth performance, blood profiles, liver weight and total protein level (TP) as well as liver gene expression in broilers fed diets with 50% and 100% naturally AFB-contaminated corn.

## MATERIALS AND METHODS

### Experimental design, broilers, and diets

The Animal Welfare Committee of China Agricultural University approved the animal care protocol used for this experiment. A total of 1200 one-day-old male broiler chicks (Cobb) with an average initial BW of 45.1±0.3 g were randomly assigned to 1 of 6 treatments in a 3×2 factorial arrangement with 3 levels of AFB-contaminated corn (0%, 50%, and 100%) and 2 absorbent levels (0% and 1%) for a 42-d study period. There were 10 replicates (cages) per treatment and 20 birds per replicate. The experiment lasted for 6 weeks, consisting of a starter phase from d 1 to 21 and a grower phase from d 22 to 42. All diets (Table 1) were formulated to meet or exceed the NRC requirements for broilers [17]. All birds were housed in an environmentally controlled facility. Diets were fed in pellet form and feed and water were provided *ad libitum* throughout the experiment. The absorbent, which consisted of equal amount of activated carbon and HSCAS, was added at the expense of corn.

All the feed samples were stored at −20°C until further analyses. Concentrations of dry matter (DM) and nitrogen (N) in the feed were analyzed in accordance with AOAC procedures [18]. The DM of the feed was determined after drying for 24 h at 103°C. The N content was determined by using a Kjeltec 2300 Analyzer (Foss Tecator AB, Hoeganaes, Sweden). The ash content was determined after the ignition of a weighed sample in a muffle furnace (Nabertherm, Bremen, Germany) at 550°C for 6 h. The ash was then digested in aqua regia (HCl/HNO_3_ mixture), and the solution was used for calcium (Ca) and phosphorus (P) determinations. Ca concentration was determined using an atomic absorption spectrophotometer (Varian’50, Varian, Palo Alto, CA, USA), and the concentration of P was determined spectrophotometrically (NanoDrop 2000c, Thermo Scientific, Delaware, MA, USA) as previously described [19]. Amino acid contents were determined, following acid hydrolysis with 6 N HCl at 110°C for 24 h, using an amino acid analyzer (Biochrom 20, Pharmacia Biotech, Cambridge, England) [20]. Before acid hydrolysis, methionine was oxidized with formic acid.

### Analysis of mycotoxins

The mycotoxins concentrations in the corn and diet were analyzed by HPLC (Agilent1100, Agilent Technologies, Santa Clara, CA, USA) according to AOAC method [18]. The detection limits of mycotoxins were 2 μg/kg for AFB_1_, 0.8 μg/kg for AFB_2_, 2.5 μg/kg for AFG_1_, 1.5 μg/kg for AFG_2_, 300 μg/kg for deoxynivalenol (DON), 100 μg/kg for zearalenone (ZEA), 30 μg/kg for T-2 toxin, 100 μg/kg for ochratoxin A (OTA), and 200 μg/kg for fumonisin B_1_ (FB_1_).

### Sample collection and measurements

The birds were weighed, and feed intake was recorded on d 1, 21, and 42, and average daily gain (ADG), average daily feed intake (ADFI), and feed-to-gain ratio (F/G) were calculated [21]. Mortality was recorded as it occurred. Both ADFI and F/G were corrected for mortality.

On d 14, 28, and 42, 2 birds from each replicate were ran domly selected from each cage and blood samples were collected from the jugular vein into a sterile syringe and stored at −4°C. Blood samples were then centrifuged at 3,000×g for 15 min and serum was separated. The levels of TP, albumin (ALB), alanine transaminase (ALT), and aspartate aminotransferase (AST), alkaline phosphatase (AKP), and γ-glutamyl transferase (γ-GT) in the serum were measured using the colorimetric method (Jiancheng Biotechnology Institute, Nanjing, China) following the kit instructions.

After blood collection, the same birds were weighed in dividually, and then sacrificed by cervical dislocation and exsanguinated. Livers were removed by trained personnel and weighed, and the relative liver weight was calculated as a percentage of BW. Liver samples were collected and then stored at −20°C until required. All liver samples were determined for TP by the colorimetric method according to the manufacturer’s instructions (Nanjing Jiancheng Bioengineering Institute, Nanjing, China).

The expression of interleukin-6 (IL-6), IL-1β, interferon-γ (IFN-γ), catalase (CAT), superoxide dismutase (SOD), glutathione peroxidase (GSH-Px), cytochrome P450 1A1 (CYP1A1), epoxide hydrolase (EH) and glutathione S-transferase (GST) in liver on d 42 were analyzed using quantitative real-time polymerase chain reaction (PCR) (MyiQ real-time PCR detection system, Bio-Rad, Hercules, CA, USA) with SYBR mix (Bio-Rad, USA). Briefly, total RNA from hepatocytes (16 samples per treatment) was extracted using TRIzol (Invitrogen, Carlsbad, CA, USA). Extracted RNA was quantified at an absorbance of 260 nm (ND-1000, Nanodrop Technologies Inc., Wilmington, DE, USA) [22]. The reverse transcription was carried out using the M-MLV reverse transcriptase (Promega, Madison, WI, USA). The standard curve was established using pooled samples, and water served as a negative control. All reactions were analyzed in duplicate and the formation of a single PCR product was confirmed by melting curves [23]. The primer sequences were designed using Primer3 software (http://primer3.wi.mit.edu; Table 2). The mRNA expression was determined from the threshold cycle for respective genes, and the expression level was calculated using the ΔΔCt method normalized using glyceraldehyde phosphate dehydrogenase (*GAPDH*) expression [24].

### Statistical analysis

A two-way analysis of variance of the data using the general linear model procedure of SAS statistical software (SAS Inst. Inc., Cary, NC, USA) was performed according to a 3×2 factorial arrangement of treatments, including contaminated corn level and absorbent level as the main effects and respective interaction. Pen was considered as the experimental unit for all measurements. Differences among treatment means were separated using the least significant difference test at p< 0.05 statistical level.

## RESULTS

### Dietary mycotoxin concentrations

The naturally mycotoxin-contaminated corn mainly contained AFB_1_ and AFB_2_, while the rest of the mycotoxins (AFG_1_, AFG_2_, DON, ZEA, T-2 toxin, OTA, FB_1_) were below the limit of detection (Table 3). Dietary AFB_1_ levels ranged from 16.7 to 84.8 μg/kg in starter diet and from 49.9 to 135 μg/kg in grower diet, and the AFB_2_ concentrations were 4.77 to 15.2 μg/kg and 10.8 to 24.8 μg/kg for the two-phase diets, respectively.

### Growth performance

During d 1 to 21, dietary AFB-contaminated diets reduced (p<0.05) BW, ADG, and ADFI, but increased F/G. Birds fed 100% AFB-contaminated diets had lower (p<0.05) ADFI, but higher (p<0.05) F/G than those fed 0% and 50% AFB-contaminated diets (Table 4). The addition of absorbent increased (p<0.05) BW, ADG, and ADFI, whereas had no effect on F/G. There were interactions (p<0.05) in BW, ADG, and ADFI between contaminated corn and absorbent.

During d 22 to 42, ADG, ADFI, and F/G were not affected by dietary contaminated corn or absorbent. No interaction was observed between contaminated corn and absorbent.

Throughout the whole experiment, birds fed 100% AFB-contaminated diets had lower (p<0.05) final BW and ADG, but higher (p<0.05) F/G compared to those fed control diets. The addition of absorbent did not affect ADG, ADFI, or F/G. There was no interaction between contaminated corn and absorbent for any measures.

### Blood profiles

On d 14, feeding AFB-contaminated diets did not affect serum TP, ALB, AST, ALT, AKP, or γ-GT levels (Tables 5, 6). The addition of absorbent increased (p<0.05) serum ALB, but reduced (p<0.05) serum ALT without any effect on TP, AST, or γ-GT. There was no interaction between contaminated corn and absorbent.

On d 28, the AFB-contaminated diets did not influence serum TP, ALB, AST, AKP, or γ-GT, whereas birds fed 100% AFB-contaminated diets had higher (p<0.05) serum ALT than those fed 0% and 50% AFB-contaminated diets. The absorbent supplementation increased (p<0.05) serum TP and ALB, but decreased (p<0.05) serum AST and AKP without any effect on ALT or γ-GT. There was no interaction between contaminated corn and absorbent except serum γ-GT (p<0.05).

On d 42, birds fed 100% AFB-contaminated diets had high er (p<0.05) serum AKP than those fed 0% and 50% AFB-contaminated diets. Dietary treatments did not affect serum TP, ALB, AST, ALT, or γ-GT. There was no interaction between contaminated corn and absorbent.

### Liver weight and total protein

The AFB-contaminated diets and absorbent had no effect on the relative liver weight throughout the entire experiment (Table 7). Feeding AFB-contaminated diets reduced (p<0.05) hepatic TP on d 28 and 42. There was no interaction between contaminated corn and absorbent.

### Liver gene expression

The AFB-contaminated diets increased (p<0.05) the expression of IL-6, CAT, SOD, and GSH-Px, but reduced (p<0.05) that of GST (Table 8). The addition of absorbent increased (p<0.05) the expression of IL-1β, CAT, SOD, CYP1A1, and GST. Significant interaction of contaminated corn and absorbent was found for the expression of CAT, SOD, and GST (p< 0.05).

## DISCUSSION

### Dietary mycotoxin concentrations

Remarkably, the contents of AFB_1_ and AFB_2_ were reduced by 60% and 40% or 68% and 51% in the starter 50% AFB-contaminated diets with adsorbent or 100% contaminated diets, respectively. In addition, the level of AFB_1_ and AFB_2_ in the grower 50% AFB-contaminated diets and 100% AFB-contaminated diets were decreased by 31% and 20% or 26% and 20%, respectively. This indicated that the absorbent (complex activated carbon and HSCAS) successfully absorbed AFB, which was consistent with previous studies [11,12]. Moreover, the results showed that the absorbent might be more effective in lower levels of AFB-contaminated diets due to the higher reduction degree. The higher AFB_1_ and AFB_2_ levels in grower diet might due to the longer storage time of the corn.

### Growth performance

Several recent studies have demonstrated that naturally AFB-contaminated grains or purified AFB could results in aflatoxicosis, which might be due to anorexia, listlessness, impaired immune and liver function, altered intestinal morphology, and inhibition of protein synthesis and lipogenesis in broilers [3–6]. The effects of corn naturally contaminated with mycotoxins on health and performance of animals may have been greater than purified mycotoxin diets [25]. Significant interactions in BW, ADG, and ADFI between contaminated corn and absorbent were found in the present study, which implied that the absorbent ameliorated aflatoxicosis for broilers. Notwithstanding, in grower phase, probably because no significant growth depression was found in broilers fed diets with contaminated corn, the adsorbent did not present an improvement in growth performance. This indicated that aflatoxicosis exerts greater negative effects on growth performance in broilers in the starter phase and the absorbent could counteracted the negative effects. As expected, low naturally AFB-contaminated diets (16 to 85 μg/kg AFB_1_ and 5 to 15 μg/kg AFB_2_) reduced BW, ADG, and ADFI, but increased F/G in broilers during starter phase, and the depression was more pronounced when the content reached 100% in the current study. Similarly, previous study reported that BW on d 21 was decreased by low levels of AFB_1_ and AFB_2_ (16 to 82 μg/kg AFB_1_ and 3 to 14 μg/kg AFB_2_) in broilers fed contaminated diets and the reduction increased with an increasing of naturally contaminated corn [4]. The increased F/G in broilers fed AFB-contaminated diets (44.5 μg/kg) was observed by previous studies [26]. Others also observed negative effects of AFB (1 to 5 mg/kg) on growth performance in broilers [5–7,27]. On the contrary, low purified AFB_1_ (50 μg/kg) did not affect growth performance in broilers [3]. The inconsistency might be attributed to the fact that naturally contaminated AFB was more toxic than purified AFB and the different protein sources and levels also would alter protein utilization and animal responses to AFB [13,28]. In grower phase, the growth performance was not affected when exposed to AFB-contaminated diets, even though the AFB_1_ and AFB_2_ concentrations were higher than that in starter phase. The results indicated that younger broilers were more susceptible to AFB than older ones. This was supported by a meta-analysis literature which found that the effect of mycotoxins on growth was greater in young broilers [7]. Feeding 100% AFB-contaminated diets reduced overall BW and ADG, but increased F/G, which was partially consistent with previous study [4]. Furthermore, the adsorbent used in present study could effectively adsorb dietary AFB_1_ and AFB_2_, and the adsorption rate was up to 68% in 100% AFB-contaminated diet. Moreover, the adsorbent supplementation improved BW, ADG, and ADFI in starter phase compared with AFB-contaminated diets and eliminated the growth reduction to control diets. The results agreed with previous studies, which also demonstrated that the activated carbon or HSCAS improved ADG and ADFI of the AFB-treated broilers [9,12,29,30].

### Blood profiles

When the liver was exposed to AFB, the hepatocytes were damaged and membrane permeability was enhanced, and the enzymes in liver (e.g. ALT, AST, and AKP) were released into the blood and consequently serum enzyme activity increased [4,6]. Feeding low levels of AFB_1_ and AFB_2_ contaminated diets had little impact on blood profiles except the serum ALT on d 28 and AKP on d 42, which were increased by dietary AFB in present study. ALT is a marker of liver injury and AKP is a signal for various liver disease states [31,32]. Therefore, the increase in serum ALT and AKP indicated that broiler livers were also damaged to some extent even when exposed to naturally low level of AFB contaminated diets for long time. In contrast, feeding high levels of AFB-contaminated diets (1.5 to 5 mg/kg) reduced the levels of TP, ALB, and GLB, which may be due to the hepatotoxic effects of AFB_1_ characterized by the inhibition of protein synthesis and impairment of carbohydrate and lipid metabolism [33,34]. This inconsistency may be due to the different AFB origins (corn naturally contaminated or inoculated with purified mycotoxins) and dosage. Nevertheless, the addition of adsorbent was able to reduce serum ALT and AKP levels in broilers fed AFB-contaminated diets. The serum AST, another marker of liver injury, was also decreased by the adsorbent on d 28. Similarly, 0.4% activated charcoal or 0.5% graphene oxide with chitosan adsorbents counteract the adverse effects of AFB-contaminated diets on serum ALT and γ-GT in broilers [9,35]. These results indicated that the adsorbent might improve cell integrity and prevent the release of liver enzymes into serum [5].

### Liver weight and total protein

It was well documented that AFB and its metabolites mainly accumulate in liver and the liver becomes the key target organ [36]. In present study, we failed to observe any statistical change in the relative weight of livers in broiler fed low levels of AFB_1_ and AFB_2_, which agreed with previous studies [3,4]. However, others observed that diets naturally contaminated with AFB_1_ and AFB_2_ increased the relative weight of livers [37–39]. The lack of effect may be caused by the low levels of AFB_1_ and AFB_2_ in the diets of our study and the differences in AFB_1_ sensitivity of the bird population assayed in each experiment [3]. It is suggested that when dietary AFB_1_ ranged from 100 to 200 μg/kg, the clinical, hematological-biochemical and histopathological changes may occur in broilers [40,41]. Besides, no significant differences were observed between broilers fed the control diet and those fed the diet containing absorbent alone, indicating that the adsorbent was inert and nontoxic in agreement with previous findings [3]. In the current study, with the increase of AFB-contaminated corn, hepatic TP levels decreased on d 28 and 42, and the reduction increased with time. The results were consistent with previous studies on broilers [34,39], illustrating that chronic aflatoxicosis would impair protein synthesis in liver and the impairment increased with time and AFB concentration.

### Liver gene expression

Although the relative weight of livers did not change, the hepatic TP levels were decreased by the AFB-contaminated diets. AFB_1_ was biotransformed into various metabolites, especially the toxic AFB_1_-exo-8,9-epoxide (AFBO), which occurred in the liver and the intestinal tract [42]. AFBO is a potent inhibitor of protein synthesis in poultry through its interaction with DNA and RNA [43]. However, literature on AFB in this regard is still scarce. A recent study evaluated the effects of AFB-contaminated diets on mRNA expression of jejunal peptide and amino acid transporters in broilers and found that a higher mRNA production is needed to increase translation process to restore possible AFB_1_ or AFBO impaired protein activities [34]. Therefore, we supposed that liver gene expression may also be modulated by the AFB-contaminated diets and hence examined the effects of AFB-contaminated diets on liver gene expression involved in inflammatory response (*IL-6*, *IL-1β*, *IFN-γ*), antioxidant function (*CAT*, *SOD*, *GST-α*) and biotransformation of AFB (*EH*, *GSH-Px*, *CYP1A1*) in response to AFB and absorbent. The cytokines (IL-6 and IL-1β secreted by activated macrophages and IFN-γ originated from T helper cells, natural killer cells, and macrophages) are released to induce inflammatory reactions and mediate the immune responses when exposed to infections in poultry [44]. The AFB-contaminated diets upregulated the expression of IL-6, which was similar with the results of previous studies in broilers fed 1 to 2 mg/kg AFB_1_ [5,16]. This indicated that the AFB_1_-contaminated diets led to hepatic inflammatory response. In addition, the reverse response to absorbent was found for IL-1β in our study. Moreover, the HSCAS can partially ameliorate the inflammation by reducing IL-6 expression in liver induced by AFB_1_ for broilers [5]. The CAT, SOD, and GSH-Px as the key enzymes of antioxidant system can scavenge free radicals generated from oxidant stress, reduce oxidative damage and maintain cell structure. The expression of CAT, SOD, and GSH-Px was upregulated by the AFB-contaminated diets and the absorbent supplementation also upregulated the expression of CAT, SOD, and GSH-Px. Similarly, previous study also observed that the SOD and GSH-Px expression in broilers was upregulated by 0.5 to 1 mg/kg purified AFB_1_, but they were not influenced by the absorbent (HSCAS) [5]. The results were not always consistent. No significant change in GSH-Px expression in response to AFB_1_ was observed [16]. AFBO can be detoxified primarily by GST enzymes by forming glutathione conjugates, or to a lesser extent by EH through a conversion to AFB_1_-dihydrodiol [45]. In our study, the AFB-contaminated diets downregulated GST expression, while the addition of absorbent increased the expression GST, which indicated that the absorbent may be effective in detoxifying. On the contrary, feeding 2 mg/kg AFB_1_-contaminated diets increased the expression of EH and GST, which indicated an increase in AFBO accumulation [5]. CYP1A1 is a member of the CYP450 enzyme family and is one of the enzymes that is responsible for activating AFB_1_. The addition of absorbent increased CYP1A1 expression, which agreed with previous study [5]. In contrast, feeding 1 mg/kg AFB_1_-contamianted diets upregulated CYP1A1 expression in broilers [16]. The inconsistency may be due to different AFB levels and origins. The current results also demonstrated that the AFB_1_-contaminated diets damaged the hepatic tissues at the molecular level.

## CONCLUSION

The naturally AFB_1_ and AFB_2_ contaminated diets depressed growth performance, especially in the starter phase and negatively affected blood profiles, while increased the expression of IL-6, CAT, SOD, and GSH-Px in liver of in broilers. The adsorbent (complex activated carbon and HSCAS) could effectively adsorb dietary AFB_1_ and AFB_2_ and alleviated aflatoxicosis by improving ADG and ADFI in the starter phase and partially restoring the negatively influenced blood profiles and hepatic gene expression in young broilers.

## Figures and Tables

**Table 1:** Diet composition (as-fed basis)

Items	Starter (d 1 to 21)	Grower (d 22 to 42)
Ingredients (%)		
Corn	56.16	59.95
Soybean meal (CP 44%)	31.50	25.45
Corn gluten meal (CP 60%)	4.65	5.02
Tallow	3.50	5.50
Limestone	1.00	1.00
Dicalcium phosphate	2.08	1.93
NaCl	0.40	0.40
L-Lys·HCl (24%)	0.20	0.20
DL-methionine (99%)	0.20	0.20
L-threonine (98.5%)	0.15	0.15
Vitamin premix1)	0.10	0.10
Trace mineral premix1)	0.10	0.10
Analytical composition		
ME (kcal/kg)2)	3,050	3,200
Crude protein (%)	22.03	19.98
Lysine (%)	1.17	1.05
Methionine (%)	0.59	0.55
Methionine+cystine (%)	0.86	0.72
Threonine (%)	0.78	0.74
Ca (%)	0.93	0.88
Total P (%)	0.72	0.67

CP, crude protein; ME, metabolizable energy.

1) Provided per kilogram of diet: vitamin A, 12,000 IU; vitamin D_3_, 3,000 IU; vitamin E, 7.5 IU; vitamin K_3_, 1.5 mg; vitamin B_1_, 0.6 mg; vitamin B_2_, 4.8 mg; vitamin B_6_, 1.8 mg; vitamin B_12_, 9 μg; folic acid, 150 μg; nicotinic acid, 10.5 mg; calcium pantothenate 7.5 mg; 100 mg Fe (FeSO_4_·H_2_O); 8 mg Cu (CuSO_4_·5H_2_O); 120 mg Mn (MnSO_4_·H_2_O); 100 mg Zn (ZnSO_4_·H_2_O); 0.3 mg Se (Na_2_SeO_3_); and 0.7 mg I (IO_3_).

2) Calculated values.

**Table 2:** Primer sequences (5′→3′) used in quantitative real-time polymerase chain reaction

Genes	Forward primer	Reverse primer
*GAPDH*	TCCTAGGATACACAGAGGACCA	CGGTTGCTATATCCAAACTCA
*SOD*	AGGGGGTCATCCACTTCC	CATTTGTGTTGTCTCCAA
*EH*	AAAGGGACAGAAGCCTGACA	CCTCCAGTGGCTCAGTGAAT
*IL-6*	GAATGTTTTAGTTCGGGCACA	TTCCTAGAAGGAAATGAGAATGC
*IL-1β*	GCATCAAGGGCTACAAGCTC	CAGGCGGTAGAAGATGAAGC
*IFN-γ*	CAAGTAATTCGGATGTAGC	GCGTTGGATTTTCAAGCC
*CAT*	GGGGAGCTGTTTACTGCAAG	TTTCCATTGGCTATGGCATT
*GSH-Px*	TTGTAAACATCAGGGGCAAA	TGGGCCAAGATCTTTCTGTAA
*GST*	GCCTGATGCACTTGCAAAA	AAAATTGCCATCAGTCTTGGT
*CYP1A1*	CACTTTCTGCCTGCTCCTG	GGTCCTTCCTCAGCTCCAG

*GAPDH*, glyceraldehyde phosphate dehydrogenase; *SOD*, superoxide dismutase; *EH*, epoxide hydrolase; *IL*, interleukin; *IFN-γ*, interferon-γ; *CAT*, catalase; *GSH-Px*, glutathione peroxidase; *GST*, glutathione S-transferase; *CYP1A1*, cytochrome P450 1A1.

**Table 3:** Concentrations of AFB_1_ and AFB_2_ in corn and diets

Items (μg/kg)	Starter (1 to 22 d)		Grower (22 to 42 d)	
	
	AFB_1_	AFB_2_	AFB_1_	AFB_2_
Contaminated corn	152	25.3	232	38.7
Control	2.3	ND	2.4	ND
50% contaminated corn	38.6	7.39	70.5	13.2
100% contaminated corn	84.8	15.2	135	24.8
50% contaminated corn+1% absorbent	16.7	4.77	49.9	10.8
100% contaminated corn+1% absorbent	28.1	7.88	100	20.1

AFB, aflatoxins; ND, not detected.

**Table 4:** Growth performance of broiler chicks fed varying contents of contaminated corn with or without absorbent

Items	BW (g/bird)			ADG (g/bird			ADFI (g/bird)			F/G		
			
	1 d	21 d	42 d	1–21 d	22–42 d	1–42 d	1–21 d	22–42 d	1–42 d	1–21 d	22–42 d	1–42 d
Dietary treatment												
Control	45.1	830^a^	2,434	37.8^a^	76.9	57.1	54.1^a^	164	104	1.44	2.13	1.83
50% contaminated corn	45.1	809^b^	2,471	36.8^b^	78.6	57.9	52.9^b^	163	105	1.45	2.08	1.82
100% contaminated corn	45.1	750^c^	2,300	34.1^c^	73.8	53.9	50.1^c^	160	102	1.48	2.16	1.88
Control+1% absorbent	45.1	822^ab^	2,457	37.4^ab^	77.8	57.6	53.9^ab^	161	103	1.45	2.07	1.80
50% contaminated corn+1% absorbent	45.1	820^ab^	2,392	37.4^ab^	74.7	56.1	54.2^a^	161	104	1.46	2.16	1.86
100% contaminated corn+1% absorbent	45.1	812^b^	2,385	36.9^b^	74.5	55.8	53.8^ab^	160	103	1.47	2.16	1.86
SEM		4.31	17.2	0.22	0.71	0.40	0.24	1.36	0.66	0.01	0.02	0.01
Main effect mean												
Contaminated corn (%)												
0	45.1	826^a^	2,445^a^	37.6^a^	77.3	57.3^a^	53.9^a^	162	104	1.44^b^	2.10	1.82^b^
50	45.1	814^b^	2,431^ab^	37.1^b^	76.7	57.0^a^	53.5^a^	162	105	1.45^b^	2.12	1.84^ab^
100	45.1	781^c^	2,342^b^	35.5^c^	74.2	54.8^b^	52.0^b^	160	103	1.48^a^	2.16	1.87^a^
Absorbent (%)												
0	45.1	796^a^	2,402	36.2^a^	76.4	56.2	52.3^a^	162	104	1.46	2.12	1.84
1	45.1	818^b^	2,411	37.2^b^	75.7	56.5	53.9^b^	161	104	1.46	2.13	1.84
Source of variation, p-value												
Contaminated corn	-	0.03	0.05	0.03	0.36	0.05	0.04	0.70	0.42	0.01	0.29	0.02
Absorbent	-	0.02	0.05	0.04	0.27	0.05	0.03	0.68	0.59	0.78	0.31	0.70
Contaminated corn×absorbent	-	0.02	0.15	0.03	0.42	0.18	0.03	0.86	0.49	0.27	0.18	0.16

Means represent 10 cages per treatment and 20 birds per cage.

BW, body weight; ADG, average daily gain; ADFI, average daily feed intake; F/G, feed-to-gain ratio; SEM, pooled standard error of the means.

a–c Means in a column with different superscripts are significantly different (p<0.05).

**Table 5:** Serum biochemical parameters of broiler chicks fed varying contents of contaminated corn with or without absorbent

Items	TP (mg/mL)			ALB (g/L)			ALT (IU/L)			AST (IU/L)		
			
	14 d	28 d	42 d	14 d	28 d	42 d	14 d	28 d	42 d	14 d	28 d	42 d
Dietary treatment												
Control	18.2	26.6	27.0	10.2	15.6abc	12.9	4.26	1.28^c^	3.08	20.1	20.2	25.6^ab^
50% contaminated corn	16.0	24.3	25.0	9.0	14.5^bc^	12.6	4.02	2.82^a^	3.61	21.5	21.2	22.1^b^
100% contaminated corn	14.6	24.4	25.3	8.2	14.0^c^	12.4	4.40	2.13abc	3.32	21.01	19.7	26.8^a^
Control+1% absorbent	17.1	26.7	24.5	10.3	16.1^ab^	11.9	3.15	2.51abc	3.05	21.9	19.5	22.8^b^
50% contaminated corn+1% absorbent	18.1	28.6	29.1	11.4	16.8^a^	13.9	2.43	3.15^a^	2.91	21.6	17.5	24.0^ab^
100% contaminated corn+1% absorbent	19.0	25.6	30.4	11.4	16.2^ab^	14.4	2.95	1.68^bc^	3.19	22.2	17.0	23.2^ab^
SEM	0.55	0.45	0.80	0.34	0.31	0.28	0.27	0.18	0.20	0.45	0.38	0.50
Main effect mean												
Contaminated corn (%)												
0	17.6	26.6	25.8	10.3	15.8	12.4	3.71	1.90^b^	3.07	21.0	19.9	24.2
50	17.0	26.5	27.1	10.2	15.6	13.2	3.23	1.90^b^	3.26	21.6	19.4	23.1
100	16.8	25.0	27.8	9.83	15.1	13.4	3.68	2.99^a^	3.25	21.6	18.3	25.1
Absorbent (%)												
0	16.3	25.1^b^	25.8	9.2^b^	14.7^b^	12.6	4.23^a^	2.08	3.33	20.9	20.4^a^	24.8
1	18.1	27.0^a^	27.9	11.0^a^	16.4^a^	13.4	2.85^b^	2.45	3.05	21.9	18.0^b^	23.4
Source of variation, p-value												
Contaminated corn	0.80	0.20	0.48	0.87	0.49	0.35	0.68	0.04	0.89	0.78	0.28	0.22
Absorbent	0.11	0.04	0.19	0.02	0.03	0.19	0.02	0.16	0.43	0.30	0.02	0.18
Contaminated corn×absorbent	0.18	0.11	0.15	0.20	0.33	0.09	0.88	0.05	0.72	0.81	0.25	0.05

Means represent 10 cages per treatment and 2 birds per pen.

TP, total protein; ALB, albumin; ALT, alanine transaminase; AST, aspartate aminotransferase; SEM, pooled standard error of the means.

a–c Means in a column with different superscripts are significantly different (p<0.05).

**Table 6:** Serum biochemical parameters of broiler chicks fed varying contents of contaminated corn with or without absorbent

Items	AKP (U/dL)			γ-GT (U/L)		
	
	14 d	28 d	42 d	14 d	28 d	42 d
Dietary treatment						
Control	368	380	93.7	13.2	31.6^a^	32.8
50% contaminated corn	340	421	264	13.0	20.0^c^	34.1
100% contaminated corn	320	373	187	13.9	25.5^cb^	35.1
Control+1% absorbent	214	317	119	14.4	24.3^bc^	33.4
50% contaminated corn+1% absorbent	388	334	247	15.2	28.8^ab^	39.7
100% contaminated corn+1% absorbent	219	175	112	15.8	27.9^ab^	43.4
SEM	27.1	26.9	23.2	0.53	0.80	1.48
Main effect mean						
Contaminated corn (%)						
0	291	348	106^b^	13.8	27.9	33.1
50	364	378	150^b^	14.1	24.4	36.9
100	270	274	256^a^	14.9	26.7	39.2
Absorbent (%)						
0	343	391^a^	182	13.4	25.7	34.0
1	274	275^b^	159	15.1	27.0	38.8
Source of variation, p-value						
Contaminated corn	0.19	0.28	0.02	0.65	0.09	0.36
Absorbent	0.39	0.21	0.19	0.80	0.07	0.22
Contaminated corn×absorbent	0.34	0.52	0.76	0.81	0.04	0.52

Means represent 10 cages per treatment and 2 birds per pen.

AKP, alkaline phosphatase; γ-GT, γ-glutamyl transferase; SEM, pooled standard error of the means.

a–c Means in a column with different superscripts are significantly different (p<0.05).

**Table 7:** Relative liver weights and liver TP of broiler chicks fed varying contents of contaminated corn with or without absorbent

Items	Relative liver weight (g/kg)			Liver TP (mg/100 mg)		
	
	14 d	28 d	42 d	14 d	28 d	42 d
Dietary treatment						
Control	26.3	21.8	26.8	9.91	8.63	9.51
50% contaminated corn	26.9	22.7	26.5	9.52	7.88	8.40
100% contaminated corn	24.7	23.4	29.7	9.26	7.60	7.63
Control+1% absorbent	25.6	21.6	25.1	9.74	8.82	8.87
50% contaminated corn+1% absorbent	24.8	22.3	27.6	9.45	7.95	8.57
100% contaminated corn+1% absorbent	26.3	22.9	26.8	9.66	8.43	8.28
SEM	0.31	0.46	0.52	0.13	0.14	0.16
Main effect mean						
Contaminated corn (%)						
0	26.0	21.7	25.9	9.82	8.72^a^	9.19^a^
50	25.9	22.5	27.1	9.49	8.02^b^	8.48^b^
100	25.5	23.1	28.2	9.46	7.92^b^	7.95^b^
Absorbent (%)						
0	26.0	22.6	27.7	9.56	8.04	8.51
1	25.6	22.3	26.5	9.62	8.40	8.57
Source of variation, p-value						
Contaminated corn	0.77	0.48	0.26	0.51	0.04	0.03
Absorbent	0.49	0.66	0.23	0.84	0.34	0.80
Contaminated corn×absorbent	0.12	0.89	0.31	0.65	0.46	0.13

Means represent 10 cages per treatment and 2 birds per pen.

TP, total protein; SEM, pooled standard error of the means.

a,b Means in a column with different superscripts are significantly different (p<0.05).

**Table 8:** Liver gene expression of broiler fed varying contents of contaminated corn with or without absorbent

Items	*IL-6*	*IL-1β*	*IFN-γ*	*CAT*	*SOD*	*GSH-Px*	*CYP1A1*	*EH*	*GST*
Dietary treatment									
Control	0.88^c^	0.78^b^	1.16	0.58^d^	0.80^d^	0.35^d^	1.10^c^	1.51	1.34^a^
50% contaminated corn	0.75^d^	0.89^b^	0.66	1.84^c^	1.43^c^	0.71^c^	0.89^c^	0.55	0.72^b^
100% contaminated corn	1.00^b^	1.04^ab^	1.46	2.37^b^	1.48^c^	2.15^a^	1.48^c^	1.01	0.52^c^
Control+1% absorbent	0.71^d^	1.54^a^	0.96	3.14^a^	2.18^a^	1.27^b^	3.11^a^	0.82	0.77^b^
50% contaminated corn+1% absorbent	0.98^b^	1.42^ab^	1.54	3.10^a^	1.82^b^	0.61^c^	2.66^ab^	0.88	1.34^a^
100% contaminated corn+1% absorbent	1.12^a^	1.31^ab^	1.62	2.52^b^	1.98^ab^	1.33^b^	2.25^b^	0.88	1.00^ab^
SEM	0.02	0.19	0.24	0.08	0.07	0.05	0.23	0.24	0.05
Main effect mean									
Contaminated corn (%)									
0	0.80^b^	1.16	1.06	1.86^b^	1.49^b^	0.81^b^	2.11	1.17	1.06^a^
50	0.87^b^	1.16	1.10	2.47^a^	1.63^ab^	0.66^c^	1.78	0.72	1.03^a^
100	1.06^a^	1.18	1.54	2.45^a^	1.73^a^	1.74^a^	1.87	0.95	0.76^b^
Absorbent (%)									
0	0.88	0.90^b^	1.09	1.60^b^	1.24^b^	1.07	1.16^b^	1.02	0.86^b^
1	0.94	1.42^a^	1.37	2.92^a^	1.99^a^	1.07	2.67^a^	0.86	1.04^a^
Source of variation, p-value									
Contaminated corn	0.03	0.34	0.67	0.02	0.04	0.02	0.48	0.22	0.02
Absorbent	0.25	0.02	0.31	0.01	0.03	0.46	0.01	0.43	0.04
Contaminated corn×absorbent	0.43	0.66	0.14	0.04	0.02	0.05	0.17	0.30	0.04

Means represent 10 cages per treatment and 2 birds per pen.

*IL*, interleukin; *IFN-γ*, interferon-γ; *CAT*, catalase; *SOD*, superoxide dismutase; *GSH-Px*, glutathione peroxidase; *CYP1A1*, cytochrome P450 1A1; *EH*, epoxide hydrolase; *GST*, glutathione S-transferase; SEM, pooled standard error of the means.

a–d Means in a column with different superscripts are significantly different (p<0.05).
